# Lin28A induces energetic switching to glycolytic metabolism in human embryonic kidney cells

**DOI:** 10.1186/s13287-016-0323-2

**Published:** 2016-05-26

**Authors:** Craig K. Docherty, Ian P. Salt, John R. Mercer

**Affiliations:** Institute of Cardiovascular and Medical Sciences, College of Medical, Veterinary and Life Sciences, University of Glasgow, Glasgow, G12 8TA Scotland, UK; BHF Glasgow Cardiovascular Research Centre, University of Glasgow, Glasgow, G12 8TA Scotland, UK

**Keywords:** Lin28, Cellular metabolism, Glycolysis, Hexokinase II, Reprogramming

## Abstract

**Background:**

Loss of a cell’s capacity to generate sufficient energy for cellular functions is a key hallmark of the ageing process and ultimately leads to a variety of important age-related pathologies such as cancer, Parkinson’s disease and atherosclerosis. Regenerative medicine has sought to reverse these pathologies by reprogramming somatic cells to a more juvenile energetic state using a variety of stem cell factors. One of these factors, Lin28, is considered a candidate for modification in the reprogramming of cellular energetics to ameliorate the ageing process while retaining cell phenotype.

**Results:**

Over-expression of Lin28A resulted in key changes to cellular metabolism not observed in wild-type controls. Extracellular pH flux analysis indicated that Lin28A over expression significantly increased the rate of glycolysis, whilst high resolution oxygen respirometry demonstrated a reduced oxygen consumption. Western blot and real-time PCR analysis identified Hexokinase II as one of the key modulators of glycolysis in these cells which was further confirmed by increased glucose transport. A metabolic switching effect was further emphasised by Western blot analysis where the oxygen consuming mitochondrial complex IV was significantly reduced after Lin28A over expression.

**Conclusions:**

Results from this study confirm that Lin28A expression promotes metabolic switching to a phenotype that relies predominantly on glycolysis as an energy source, while compromising oxidative phosphorylation. Mechanisms to augment regulated Lin28A in age related pathologies that are characterised by mitochondria dysfunction or in differentiated and aged post-mitotic cells is the future goal of this work.

**Electronic supplementary material:**

The online version of this article (doi:10.1186/s13287-016-0323-2) contains supplementary material, which is available to authorized users.

## Background

Regulation of cellular metabolism holds great potential to intervene in the progression of human disease and ageing [1]. Research suggests that loss of a cell’s energetic capacity during ageing and age related pathologies can be causative and therefore amenable to intervention [2–4]. Regenerative medicine has identified stem cell factors capable of fundamentally reorganising cellular metabolism and cell phenotype which may further be able to reprogram metabolism within the context of cancer, cardiovascular disease and kidney disease [5–7]. However, to provide a new therapeutic avenue in differentiated somatic cell types their mechanism of action needs to be clarified.

Lin28 is an RNA binding protein that positively regulates embryogenesis timing and progenitor self-renewal [8]. Recognised as one of the key pluripotency markers it exists as two conserved paralogs, Lin28A and Lin28B, which are highly expressed during embryogenesis, where it promotes cell growth and maintenance of a more juvenile energetic phenotype [9]. Both homologs repress the Let-7 (lethal-7) family of regulatory microRNAs (miRNA) [10, 11] required for terminal cell differentiation by inhibiting the drosha and dicer microprocessor complexes required for the production of mature miRNA [1]. Most mature cells observe down regulation of Lin28 that occurs during differentiation with a concurrent increase in expression of mature Let7 miRNAs to maintain terminal cell fate [10].

Metabolomic profiling confirms promotion of an embryonic like bio-energetic state that leads to an enhanced metabolism from glycolysis [1, 10, 12]. Enhanced glucose metabolism is a key hallmark of functional Lin28 within these cells. Indeed, Lin28 over expression in both in vitro and in vivo models demonstrates increased glucose metabolism via increased glucose uptake and an increase in the glycolytic enzyme pyruvate dehydrogenase kinase 1 PDK1 [5]. Over-expression of Lin28 also results in increased tissue repair and an improved glucose tolerance [13]. However, unregulated Lin28 expression is also implicated in human malignancies through increases in glycolytic metabolism and cellular proliferation [13–15] intimating that unregulated Lin28 may be oncogenic. Studies on human embryonic stem cells have also suggested that the Lin28/Let 7 axis interacts with multiple mitochondrial enzymes suggesting further effects on oxidative phosphorylation [16]. Therefore, modification of Lin28 expression may have multiple roles in tissue regeneration and reprogramming metabolism.

Although many in vitro studies have elucidated the role of Lin28 in glycolysis, the exact role Lin28 plays in regulating oxidative phosphorylation is unknown. This study outlines key effects Lin28 has on mitochondrial activity and demonstrates further specific effects Lin28 has on glycolysis.

## Methods

### Cell culture

Cells were maintained in high glucose (4.5 g/L) Dulbecco’s Modified Eagle’s Medium (DMEM) containing 15 % (v/v) foetal bovine serum, 2 mM L-glutamine, 1 mM pyruvate, 100 U/ml penicillin and 100 mg/ml streptomycin. Lin28A cells were supplemented with 10 μg/ml of the selection reagent Basticidin-S hydrochloride (Sigma). Lin28A over-expressing human embryonic kidney (HEK) 293 cells were obtained from Amsbio, UK. Non-transfected HEK293 cells were obtained from Professor Andrew Baker (University of Edinburgh).

### Quantitative real-time PCR

For gene expression experiments cells were grown in six-well plates until they reached 90–100 % confluency. QIAzol lysis reagent was added to wells and cells were scraped into RNAse free micro-centrifuge tubes and stored at -80 °C. RNA species were then extracted with the miRNeasy® mini kit (Qiagen), quantified using a nanodrop spectrophotometer and normalised. RNA was then reverse transcribed to cDNA and miRNA using a TaqMan® reverse transcription kit and an miRNA reverse transcription Kit, respectively (Applied Biosystems). For miRNA, the amplification step was performed using specific TaqMan® miRNA probes. Real-time PCR was performed using the Applied Biosystems 7900 HT real-time PCR system following the manufacturer’s instructions. Specific TaqMan® primer-probes were purchased from ThermoFisher (Additional file 1: Table S1) and analysed using the delta CT method.

### Western blotting

Cells were lysed in 1 % (w/v) lauryl maltoside detergent (Abcam) in PBS and sonicated. A total of 20 μg of protein lysates was fractionated on 4–12 % gradient polyacrylamide gels and transferred to nitrocellulose membranes (Amersham). Membranes were then blocked in a 1:1 mix of SEA block (Thermo-Fisher) and Tris-buffered saline containing 0.1 % (v/v) tween 20 (TBST). Membranes were incubated overnight at 4 °C with the relevant primary antibody. Membranes were then washed in TBS/T and incubated with fluorescent secondary antibody (1:15000) for 2 hours. Membranes were then transferred to the LI-COR Odyssey-Sa infrared imaging system for visualisation and quantification. Densitometric analysis was then performed on LI-COR image studio light (version 5.2). All primary antibody dilutions are outlined in Additional file 1: Table S2.

### Extracellular flux analysis

Extracellular acidification rate (ECAR) was evaluated using the Seahorse XF24 analyser (Seahorse Bioscience, MA, USA). Briefly, 5 × 10^4^ cells were seeded in 24-well seahorse plates 24 hours prior to the experimental run. On the day of the experiment media were changed to glucose and sodium pyruvate-free XF assay media and transferred to the XF24 analyser (#102365-100; Seahorse Bioscience, MA, USA). ECAR was determined after the sequential addition of D-glucose (10 mM final), oligomycin (1 μM) and 2-deoxyglucose (2-DG; 100 mM). All compounds were purchased from Sigma, USA.

### High resolution oxygen respirometry

Cells were trypsinized and diluted in MiR05 respiration buffer (0.5 mM EGTA, 3 mM MgCl_2_, 60 mM potassium lactobionate, 20 mM taurine, 10 mM KH_2_PO_4_, 20 mM HEPES-KOH pH 7.1, 110 mM sucrose, and 1 g/L fatty acid free BSA) (Oroboros, Innsbruck, Austria) to 0.5 × 10^6^ per ml and 2 ml of cells were transferred to the O2K Oroborus respirometer (Oroboros, Innsbruck, Austria). Oxygen consumption rate (OCR) was determined after the sequential addition of oligomycin (1 μM), carbonyl cyanide m-chlorophenylhydrazone (CCCP; 1-3 μM), rotenone (1 μM) and antimycin (1 μM).

### Cell proliferation analysis

Cells were transferred to 12-well plates at a seeding density of 1 × 10^4^ cells/ml. Cell numbers from three wells were then counted every 24 hours over a 4-day period using a Hemacytometer.

### Glucose transport assay

2-[^3^H] Deoxy-D-glucose transport was measured in cells grown on12-well plates. Briefly, cells were incubated in phosphate-buffered Krebs solution (KRP buffer; 128 mM NaCl, 4.7 mM KCl, 5 mM NaH_2_PO_4_, 1.2 mM MgSO_4_, 2.5 mM CaCl_2_, 5 mM glucose and 0.1 % BSA) for 30 mins at 37 °C then transferred to glucose-free KRP for a further 30 mins. Glucose transport was initiated by addition of 2-[^3^H] deoxy-D-glucose (final concentration 25 μmol/L and 1 μCi/ml) to each well. The mixture was then incubated for 5 min. Non-specific association of radioactivity was determined by prior addition of cytochalasin B (10 μmol/l). Cell culture plates were then immersed in ice-cold PBS and allowed to dry before addition of 1 % (v/v) Triton X-100. Samples were added to scintillation vials 24 hours later and radioactivity was measured using a Beckman LS6500 scintillation counter.

### Data analysis

All data are presented as mean ± standard error of the mean (SEM) where appropriate. P values were calculated using student’s unpaired t-tests (two-tailed distribution). Statistical significance was displayed as P < 0.05 (one star), P < 0.01 (two stars) or P < 0.001 (three stars). All analysis was performed on Microsoft Office Excel 2007 or Graphpad Prism (v 5). All graphs were produced on Graphpad Prism (v 5).

## Results

### Characterisation of Lin28 in HEK293 cells

**Fig. 1:**
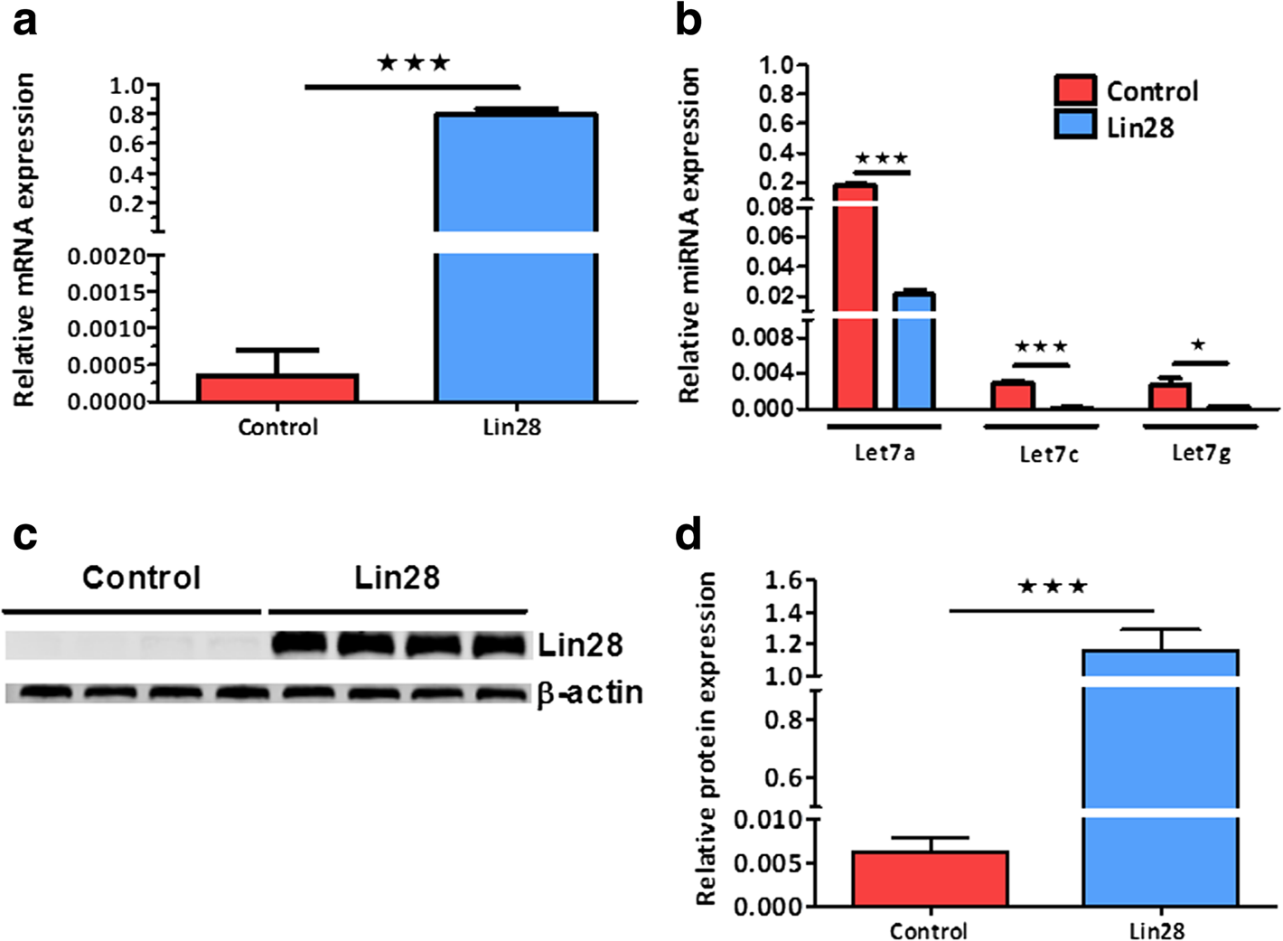
Characterisation of Lin28A over-expression in HEK 293 cells. Real-time PCR revealed that Lin28 mRNA expression levels were increased in Lin28A over-expressing cells, n = 3 (**a**). Conversely, Let 7 a, c and g miRNA expression levels were decreased in Lin28 over-expressing cells, n = 3 (**b**). Western blot analysis (**c**) confirmed that Lin28 protein levels were also increased in over-expressing HEK cells (**d**), n = 4. GAPDH and U6 were used as housekeeping genes for mRNA and miRNA expression, respectively. Student’s unpaired t-tests were used to compare between experimental groups. Data are represented as mean ± SEM. *HEK* human embryonic kidney, *miRNA* microRNA

To identify the effect of over-expression of Lin28 in our cell lines, we first examined mRNA expression using q-PCR (Taqman) (Fig. 1a). Increased Lin28A mRNA resulted in a concomitant downregulation of the dominant Let-7 miRNA transcripts let 7a (P < 0.001), c (P <0.001) and g (P < 0.05) (Fig. 1b). To examine the functional effects of Lin28 over-expression, Lin28 protein levels were also assessed (Fig. 1c). HEK293 cells overexpressing Lin28 exhibited significantly up-regulated protein expression compared to wild-type controls (P < 0.001) (Fig. 1d).

### Lin28 over-expression induces glycolytic ‘switching’

**Fig. 2:**
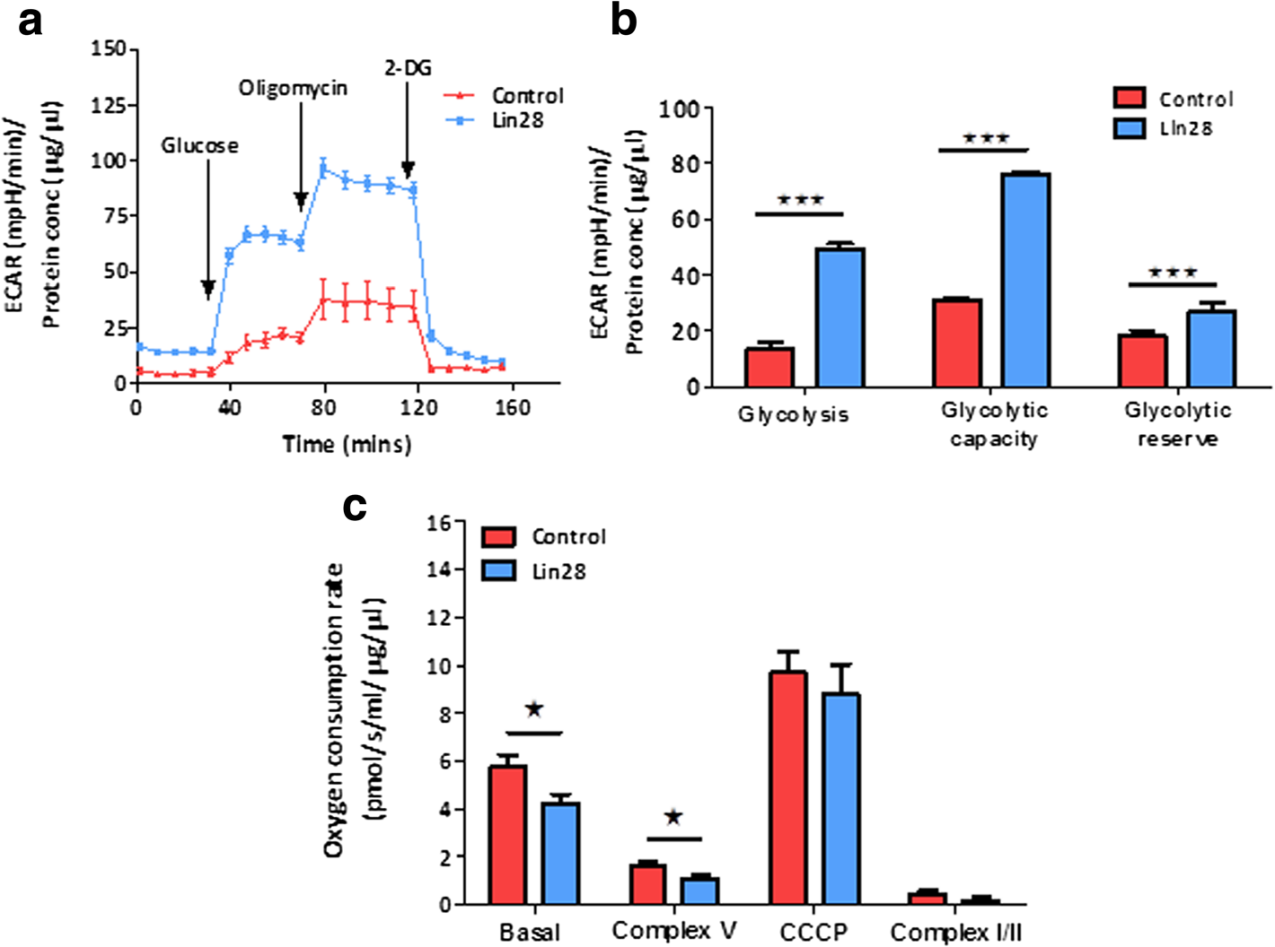
Lin28A promotes energetic switching to a more glycolytic phenotype. Extracellular flux analysis using the Seahorse XF24 bio-analyser demonstrated that ECAR was significantly increased in Lin28A over-expressing cells after addition of 10 mM glucose (glycolysis). ECAR was also increased after subsequent addition of 1 μM oligomycin (glycolytic capacity) and 100 mM 2-DG (glycolytic reserve) (**a** and **b**). Conversely, high resolution oxygen respirometry showed that even without stimulation basal oxygen consumption was reduced in over-expressing cells. No changes in maximal respiration (after addition of 1 μM CCCP) or spare capacity (after addition of 1 μM antimycin and 1 μM rotenone) were observed (**c**). Student’s unpaired t-tests were used to compare between experimental groups. Data are represented as mean ± SEM, n = 5 for all data points. *ECAR* extracellular acidification rate, *2-DG* 2-deoxyglucose, *CCCP* carbonyl cyanide *m*-chlorophenyl hydrazone

Over-expression of Lin28 significantly increased the maximum glycolytic rate within HEK293 cells (Fig. 2a). The extracellular acidification rate (ECAR) is a measure of milli-pH change attributed to accumulation of glycolytic pyruvate acid and is a measure of glycolytic rate (Seahorse Technologies ®). We found that Lin28 enhanced glycolysis (P <0.001), glycolytic capacity (P <0.001) and glycolytic reserve (P <0.001, Fig. 2b) after stimulation with glucose and inhibiting oxidative phosphorylation. However, basal oxidative metabolism, measured by oxygen flux (OCR) (P < 0.05) was significantly reduced in Lin28 over-expressers compared to the un-transfected HEK293 cells (Fig. 2c). Interestingly, no significant difference in maximal respiration was observed between the two cell types after respiratory uncoupling of the mitochondrial membrane potential (ΔΨm) with carbonyl cyanide *m*-chlorophenyl hydrazone CCCP (1μg/ml).

### Lin28 over-expression increases cellular proliferation and glucose uptake

**Fig. 3:**
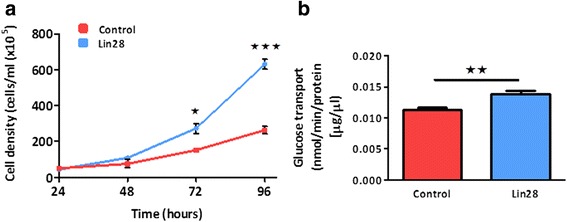
Lin28A increases cellular proliferation and glucose transport in HEK 293 cells. Proliferation studies revealed that cell density was significantly increased at 72 and 96 hour time points in Lin28A over-expressing cells compared to non-transfected (blank) HEK cells, n = 3 (**a**). Radio-labelled glucose transport assay revealed that 2-deoxyglucose transport was also significantly increased in the Lin28A over-expressing cells (P < 0.01, n = 6) (**b**). Student’s unpaired t-tests were used to compare between experimental groups. Data are represented as mean ± SEM. *HEK* human embryonic kidney

In order to assess the functional consequences of Lin28 over-expression, cellular proliferation and glucose transport were assessed. Increased proliferation of cells over-expressing Lin28A was observed at 72 hours (P < 0.05) and 96 hours (P < 0.001, Fig. 3a). 2-Deoxyglucose uptake was also demonstrated to be significantly increased in cells over-expressing Lin28 (P < 0.01, Fig. 3b).

### Lin28 alters several glycolytic enzymes

**Fig. 4:**
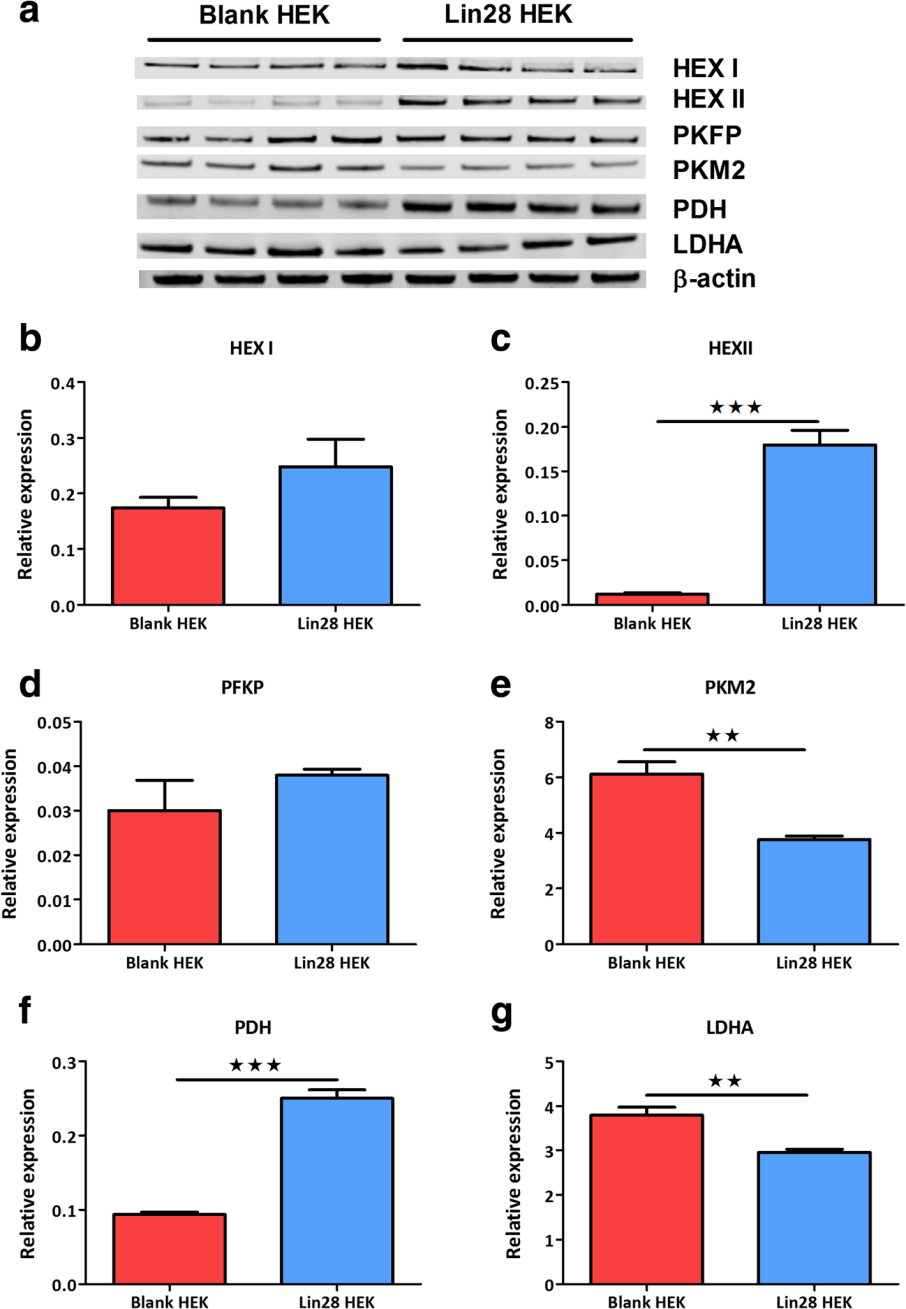
Lin28A over-expression alters several glycolytic enzymes. Densitometry analysis of Western blot data (**a**) showed that hexokinase II (**c**) and PDH (**f**) expression was significantly increased in Lin28A over-expressers. However, PKM2 (**e**) and LDHA (**g**) were significantly decreased in Lin28A over expressers. No change was observed in Hex I (**b**) or PFKP (**d**). (Student’s unpaired t-tests were used to compare between experimental groups. Data are represented as mean ± SEM, n = 4. *PDH* pyruvate dehydrogenase, *PKM2* pyruvate kinase muscle isoform 2, *LDHA* Lactate dehydrogenase A, *Hex I* hexokinase I, *PFKP* pyruvate fructose kinase phosphate

In assessing the mechanism of Lin28A’s effects in metabolic switching we examined several key enzymes involved in the glycolytic pathway using Western blotting. Hexokinase II (Hex II) was significantly increased in Lin28 over-expressing cells (P < 0.001, Fig. 4c) as was pyruvate dehydrogenase (PDH, P < 0.001, Fig. 4f). Pyruvate kinase muscle isoform 2 (PKM2) was significantly decreased in Lin28A over-expressers (P <0.01). Lactate dehydrogenase A (LDHA) was also significantly decreased in Lin28 cells (P < 0.01, Fig. 4g). No differences were observed in hexokinase I (Hex I), pyruvate fructose kinase phosphate (PFKP) compared to age and passage matched controls.

### Complex IV is reduced in Lin28A over-expressing cells

**Fig. 5:**
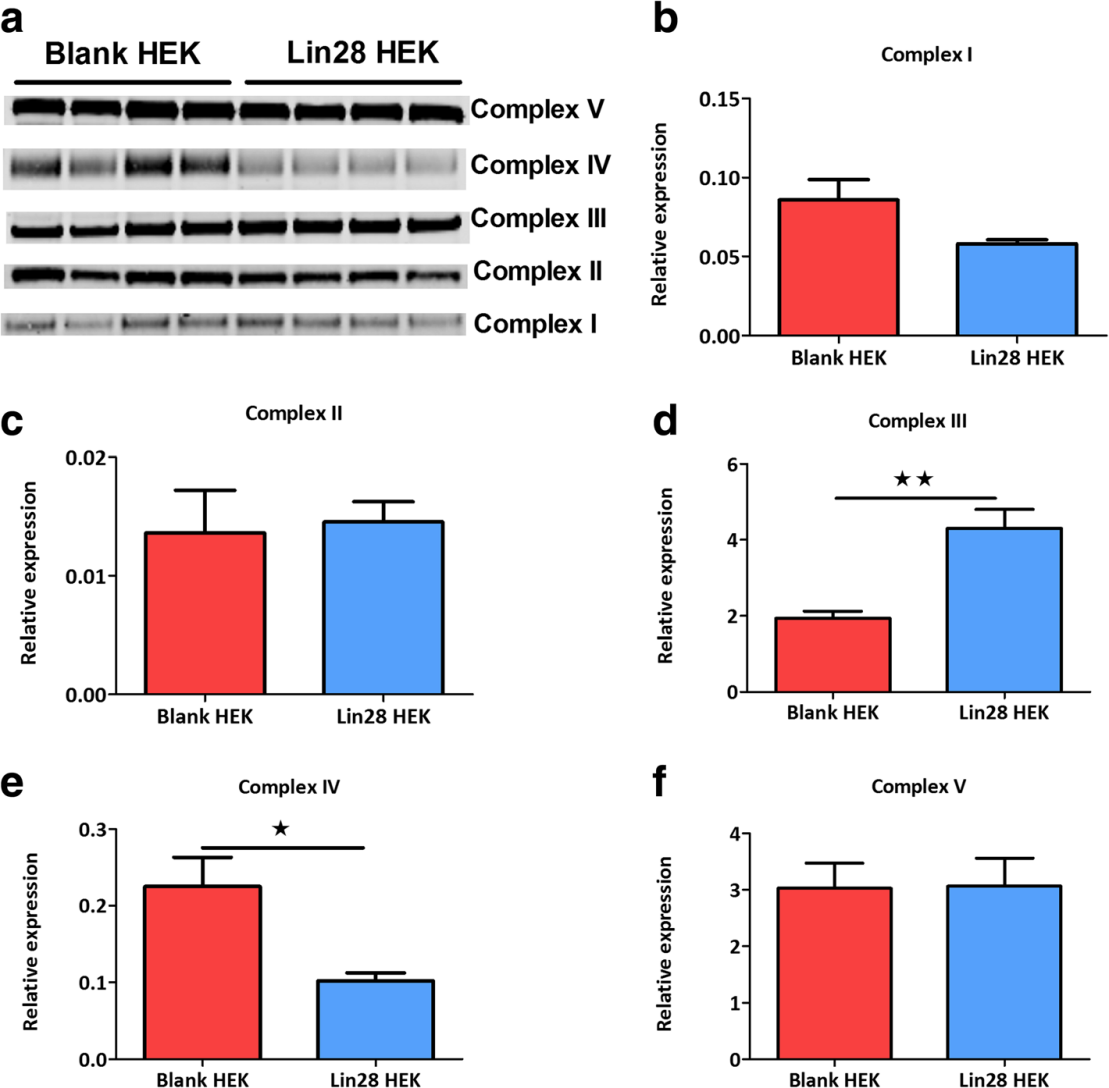
Lin28A over-expression has specific effects on mitochondrial complexes III and IV. Densitometric analysis of Western blot data (**a**) revealed no difference in complex I (**b**), II (**c**) or Complex V (**f**) but mitochondrial complex III significantly increased in Lin28A cells (**d**). A significant reduction in complex IV protein expression was also observed (**e**). Student’s unpaired t-tests were used to compare between experimental groups. Data are represented as mean ± SEM, n = 4

The mitochondrial respiratory complex antibody cocktail (Abcam ab110413) was used to assess any differences in the protein levels of individual respiratory complexes and normalised to nuclear encoded citrate synthase (CS) or manganese superoxide dismutase (MnSOD). In Lin28A over-expressing cells complex IV was significantly decreased (P < 0.05, Fig. 5e); however, complex III was significantly increased in the over expressing cells (P < 0.001). No differences were observed in complexes I, II and V when compared to age matched controls.

### Lin28A also has specific transcriptional effects

**Fig. 6:**
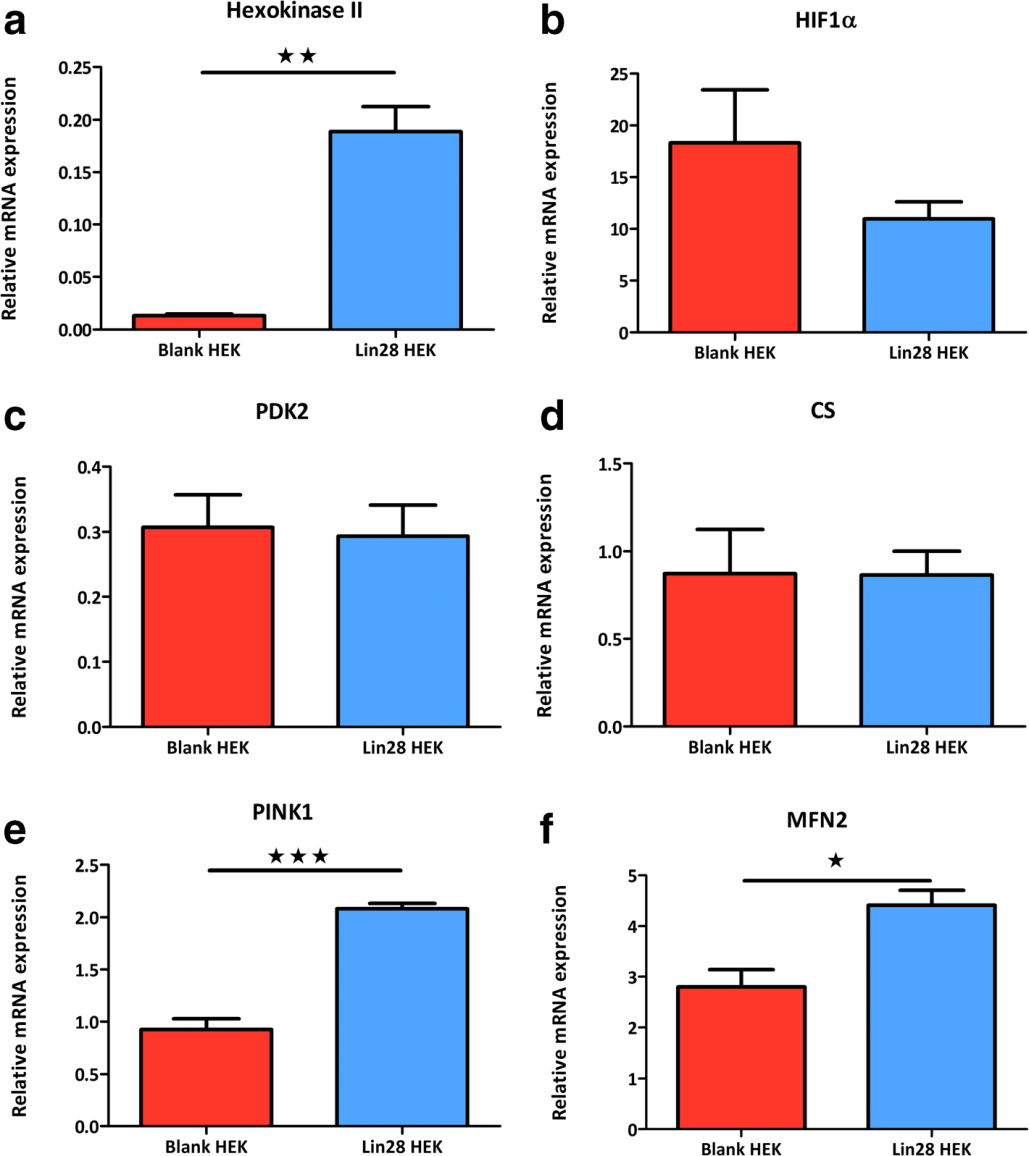
Lin28A over-expression has various effects on mRNA transcription. Real-time PCR analysis revealed over-expression of Lin28A increased hexokinase II mRNA expression (**a**). No significant differences were observed in expression levels of HIF1α (**b**), PDK2 (**c**) or citrate synthase (**d**). Mitophagy markers PINK1 and MFN2 were significantly increased in Lin28A over-expressing cells (**e** and **f**). 18S was used as a housekeeper gene for normalisation purposes. Student’s unpaired t-tests were used to compare between experimental groups. Data are represented as mean ± SEM, n = 3 for each group. *HIF1α* hypoxia inducible factor 1 α, *PDK2* pyruvate dehydrogenase kinase 2, *PINK1* PTEN-induced kinase1, *MFN2* mitofusin 2, *CS* citrate synthase

Several markers known to be involved in glycolytic switching were also assessed at the transcriptional level using real-time quantitative PCR. Similar to protein expression, Hex II mRNA was significantly increased in Lin28A over expressing cells (P > 0.01, Fig. 6a); however, no differences in pyruvate dehydrogenase kinase 2 (PDK2) or hypoxia inducible factor 1 α (HIF1α) mRNA expression was observed (Fig. 6b and c, respectively). No significant difference was observed in the nuclear encoded mitochondrial marker citrate synthase (CS) between cell types. Interestingly, the mitophagy markers phosphatase and tensin homolog induced putative kinase 1 (PINK1) and mitofusin 2 (MFN2) were significantly increased in Lin28A over-expressing cells (P < 0.001, Fig. 6e and P < 0.05, Fig. 6f, respectively).

## Discussion

In the present study we have identified the distinct metabolic changes occurring in the reprogramming of a transgenic human kidney line using over-expression of Lin28A and concomitant down regulated expression of Let7-g. Notably, this included a metabolic ‘switching’ from an energetic state relying predominantly on oxidative phosphorylation to a more glycolytic phenotype, whilst aerobic culture conditions remained constant. Indeed, this ‘metabolic reprogramming’ to a more glycolytic phenotype has been described in detail in previous studies [1, 5, 13]. However, we have outlined several new mechanisms not previously identified that may contribute to Lin28’s capacity to increase glycolysis and enhance a cell’s metabolic and mitotic performance. We have demonstrated an increase in Hex II at both the transcriptional and protein levels as well as an increase in glucose transport. These effects demonstrate that Lin28’s ability to increase Hex II is a crucial step in glycolytic switching. Hex II is the most active isoform of the hexokinase family [17] and increased intracellular Hex II activity is regarded as the rate limiting step in maintaining the glucose concentration gradient by reducing intracellular glucose through phosphorylation of glucose to glucose-6-phosphate [18]. In these cells Hex II appears the key driver of glucose transport which is confirmed by a combination of phosphorylation and increased glucose uptake, which is predicted to occur through the glucose transporters, such as GLUT-1, -2 and GLUT-4 [5, 18–20], as well as the sodium glucose co-transporter 2 (SGLT-2) [21]. Interestingly, an increase in PDH and a decrease in LDHA in Lin28A cells confirmed a preference for pyruvate decarboxylation of pyruvate to produce acetyl-CoA rather than conversion of pyruvate to lactate. This reduction in LDHA would further increase the amount of pyruvate for conversion to acetyl-CoA. Of course, acetyl-CoA can be used a substrate in oxidative phosphorylation and may be a compensatory response to an increase in glycolytic rate. A reduction in LDHA is also intriguing because increased LDHA is known to be a driver of glycolysis and subsequently cancer progression, a characteristic previously attributed to Lin28 over expression [22]. Interestingly, PKM2 protein expression was decreased in Lin28A over-expressing cells. This was surprising as much work has outlined the role of PKM2 as a molecular driver for glycolysis in many cancers [23, 24]. Indeed, results from this study suggest that, at least in HEK cells, PKM2 does not contribute significantly to glycolysis. Indeed, in our model we did not investigate the nature of the specific isoforms of PKM. These couldhave significant consequences for glycolytic metobolism [25], certainly this would require closer scrutiny in relation to Lin28 expression. HIF1α is also known to be important in the progression of several cancers, including renal cell carcinoma; therefore, HIF1α expression was assessed [5, 26]. Results from this study showed that Lin28’s increase in glycolysis did not appear to be HIF1α-dependent and is consistent with previously published data [5]. Equally, the oncogene c-myc is a known driver of Lin28, but was not overexpressed in control cell lines or in response to Lin28 (Additional file 1: Figure S1). This, combined with a notable reduction in other known cancer-related proteins such as PDK and LDHA, suggests Lin28’s role as an oncogene itself may be overstated and may be amenable as an in vitro therapeutic target in certain circumstances. Indeed, the mechanism of how up-regulation of stem cell factors, such as Lin28 effectors, can ameliorate the reliance on ox-phos through elegant Let7 anti-miR work corroborates this link [1].

Building on these findings we now demonstrated mRNA transcripts of several mitochondrial-related proteins have yielded additional intriguing results. The mitophagy marker PINK1 (PTEN-induced kinase1) [27] and its downstream effector Mitofusin 2 (MFN2) were significantly increased in Lin28 over expressing cells. This suggests a novel role for Lin28 in promoting energetic switching to a more glycolytic phenotype through mediating enhanced mitochondrial recycling which may underpin the specific effects of Lin28 on oxidative phosphorylation that include a decrease in basal oxygen consumption using a high resolution oxygen respirometry. Further analysis revealed a reduction in the oxygen consuming complex IV proteins which may account for the reduction in basal oxygen consumption and adding further weight to the notion that reduced oxidative metabolism in these cells is driven by Lin28. A reduction in oxygen consumption has been noted in various Lin28 studies utilising cancer cell lines, such as Hep3B cells (Ma *et al.,* 2014). However, in contrast, mouse embryonic fibroblasts (MEF) isolated from mice where Lin28 was over-expressed showed an increase in oxygen consumption rate [13], suggesting Lin28A over expression may have distinct effects depending on cell type [6, 7, 13]. The remarkable metabolic plasticity we show here suggests that use of synthetic targeted nucleases, such as inducible clustered regularly interspaced short palindromic repeats (CRISPR), or age related inducible expression vectors, may eventually be able to augment favourable changes in cells and tissues of choice.

## Conclusions

Results from this study confirm that augmenting Lin28A expression in differentiated epithelial lineages has the potential to reprogram cellular energetics through increasing Hex II expression and activity. A number of degenerative pathologies could be potential beneficiaries of this cellular reprogramming [28–30].

### Consent for publishing

All authors offer their full consent in the publishing of this manuscript. Consent forms are available on request.

### Availability of data

The University of Glasgow, our approved data repository, provides a comprehensive data management and freely available service which supports the principles of open access details of which can be found here:

http://www.gla.ac.uk/services/datamanagement/lookingafteryourdata/preservation/repositories/.

## Additional file

Additional file 1: Supplementary information. (DOC 164 kb)

## Abbreviations

anti-miR: anti microRNA
CCCP: carbonyl cyanide *m*-chlorophenyl hydrazone
CRISPR: clustered regularly interspaced short palindromic repeats
HEK: human embryonic kidney
HEX II: hexokinase 2
LDHA: lactate dehydrogenase
Let-7: lethal-7 family of regulatory
MEF: mouse embryonic fibroblasts
MFN2: mitofusin 2
miRNA: microRNAs
PDH: pyruvate dehydrogenase
PINK1: PTEN-induced kinase1
SGLT-2: sodium glucose co-transporter
