# Supplementary material for: Lin28A induces energetic switching to glycolytic metabolism in human embryonic kidney cells
Source: Stem Cell Res Ther. 2016 May 26;7:78. doi: 10.1186/s13287-016-0323-2 (PMC4882770; doi:10.1186/s13287-016-0323-2)
Supplement: Additional file 1: — Supplementary information. (DOC 164 kb) [file 13287_2016_323_MOESM1_ESM.doc]

**Supplementary information**

**
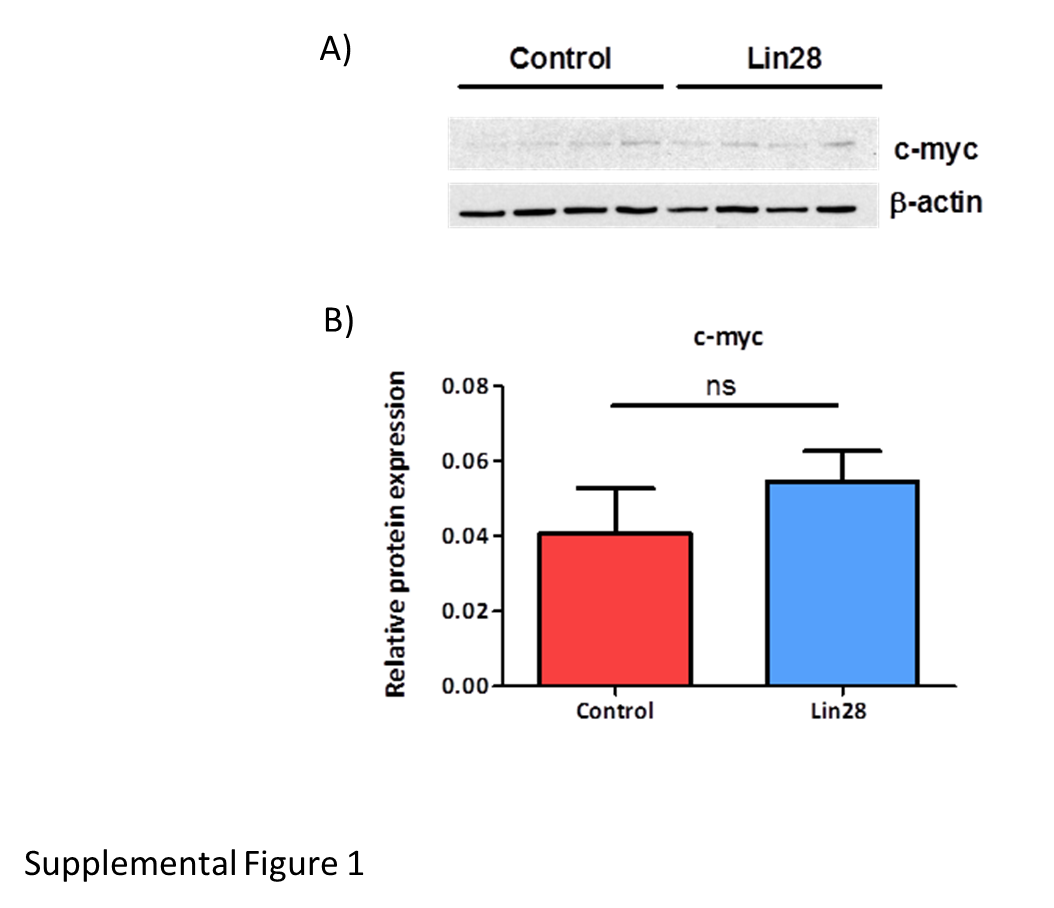
**

**S1)** western blot for C-MYC expression in control HEK and Lin28 overexpressing cells, expression normalised to beta actin **A)** Relative protein expression of C-MYC in control and Lin28 overexpressing cells **B)**

| **Gene/miRNA transcript** | **Species** | **Assay ID/product number** |
| --- | --- | --- |
| hsa-let-7a | Human | hsa-let-7a/ 000377 |
| hsa-let-7c | Human | hsa-let-7c/ 000379 |
| hsa-let-7g | Human | hsa-let-7g/ 002282 |
| Lin28A | Human | Hs01552403_g1 |
| HexII | Human | Hs00606086_m1 |
| Lin28A | Human | Hs01552403_g1 |
| HIF1α | Human | Hs00153153_m1 |
| PDK2 | Human | Hs00176865_m1 |
| MFN2 | Human | Hs00208382_m1 |
| PINK1 | Human | Hs00260868_m1 |
| CS | Human | Hs00830726_m1 |
| 18S | Human | 4319413E |
| GAPDH | Human | Hs02758991_g1 |
| U6 | Human | U6 snRNA/ 001973 |

**Table S1.** TaqMan ® gene expression assays purchased from AppliedBiosystems (ThermoFisher). HexII, Hexokinase II; HIF1α, hypoxia inducible factor 1 α; PDK2, pyruvate dehydrogenase kinase 2; MFN2, mitofusin 2; PINK1, PTEN-induced putative kinase 1; CS, citrate synthase and GAPDH, Glyceraldehyde-3-Phosphate Dehydrogenase.

| **Primary Antibody** | **Host species (clone)** | **Source (product number)** | **Dilution** |
| --- | --- | --- | --- |
| Lin28A | Rabbit polyclonal | Cell signalling (#3978) | 1:1000 |
| HexI | Rabbit polyclonal | Cell signalling (#8337) | 1:1000 |
| Hex II | Rabbit polyclonal | Cell signalling (#8337) | 1:1000 |
| PFKP | Rabbit polyclonal | Cell signalling (#8337) | 1:1000 |
| PKM2 | Rabbit polyclonal | Cell signalling (#8337) | 1:1000 |
| PDH | Rabbit polyclonal | Cell signalling (#8337) | 1:1000 |
| LDHA | Rabbit polyclonal | Cell signalling (#8337) | 1:1000 |
| C-MYC | Mouse monoclonal | Santa Cruz  (9E10) | 1:200 |
| β-actin | Mouse monoclonal | Thermo-Fisher (MA5-15739) | 1:1000 |

**Table S2**. Specifications and sources of primary antibodies used in Western blotting experiments. Hex I, Hexokinase I; Hex II, Hexokinase II; PFKP, phospho-fructose kinase phosphate; pyruvate kinase muscle isoform 2, PKM2; pyruvate dehydrogenase, PDH and lactate dehydrogenase.
